# Androgen Receptor Signaling Pathway in Prostate Cancer: From Genetics to Clinical Applications

**DOI:** 10.3390/cells9122653

**Published:** 2020-12-10

**Authors:** Gaetano Aurilio, Alessia Cimadamore, Roberta Mazzucchelli, Antonio Lopez-Beltran, Elena Verri, Marina Scarpelli, Francesco Massari, Liang Cheng, Matteo Santoni, Rodolfo Montironi

**Affiliations:** 1 Medical Oncology Division of Urogenital and Head and Neck Tumours, IEO, European Institute of Oncology IRCCS, 20141 Milan, Italy; gaetano.aurilio@ieo.it (G.A.); elena.verri@ieo.it (E.V.); 2 Section of Pathological Anatomy, School of Medicine, United Hospitals, Polytechnic University of the Marche Region, 60126 Ancona, Italy; a.cimadamore@staff.univpm.it (A.C.); r.mazzucchelli@staff.univpm.it (R.M.); m.scarpelli@univpm.it (M.S.); 3 Department of Surgery, Cordoba University Medical School, 14071 Cordoba, Spain; em1lobea@gmail.com; 4 Division of Oncology, S. Orsola-Malpighi Hospital, 40138 Bologna, Italy; francesco.massari@aosp.bo.it; 5 Department of Pathology and Laboratory Medicine, Indiana University School of Medicine, Indianapolis, IN 46202, USA; liang_cheng@yahoo.com; 6 Oncology Unit, Macerata Hospital, 62100 Macerata, Italy; mattymo@alice.it

**Keywords:** prostate cancer, androgen receptor, AR-V7, AR variants, AR antagonists, AR resistance

## Abstract

Around 80–90% of prostate cancer (PCa) cases are dependent on androgens at initial diagnosis; hence, androgen ablation therapy directed toward a reduction in serum androgens and the inhibition of androgen receptor (AR) is generally the first therapy adopted. However, the patient’s response to androgen ablation therapy is variable, and 20–30% of PCa cases become castration resistant (CRPCa). Several mechanisms can guide treatment resistance to anti-AR molecules. In this regard, AR-dependent and -independent resistance mechanisms can be distinguished within the AR pathway. In this article, we investigate the multitude of AR signaling aspects, encompassing the biological structure of AR, current AR-targeted therapies, mechanisms driving resistance to AR, and AR crosstalk with other pathways, in an attempt to provide a comprehensive review for the PCa research community. We also summarize the new anti-AR drugs approved in non-metastatic castration-resistant PCa, in the castration-sensitive setting, and combination therapies with other drugs.

## 1. Introduction

Prostate cancer (PCa) is the fifth leading cause of cancer death worldwide, and its incidence has been increasing in the U.S. since 1950, apparently related to an overall increase in life expectancy [1]. Localized PCa is usually indolent with a 5-year survival rate of nearly 100%. The 5-year survival rate drops down to 30% in metastatic PCa patients. Around 80–90% of PCa cases are dependent on androgen at initial diagnosis; hence, androgen ablation therapy directed toward a reduction in serum androgens and the inhibition of androgen receptor (AR) is generally the first therapy adopted. However, patient response to androgen ablation therapy is variable, and 20–30% of PCa patients experience progression even when the testosterone level in their blood is below 20 ng/dl. In this phase, the tumor becomes castration-resistant (CRPCa). Several studies have evidenced a role of AR in the development of resistance to castration therapies.

PCa development and growth are controlled by testosterone and 5α-dihydrotestosterone (DHT), which exert their biological effects through binding to AR. Upon binding the hormone ligand, the receptor dissociates from the accessory proteins, translocates into the nucleus, dimerizes, and then binds to androgen response element (ARE) sited in the promoter regions of genes involved in cellular proliferation and evasion of apoptosis [2,3,4]. Moreover, as with other steroids, androgens can also exert very rapid actions by triggering signaling circuits initiated outside the nucleus. This rapid effect takes place through AR interaction with a number of signaling or scaffold molecules and has been proven to be involved in cell cycle control, proliferation, migration, and nuclear exclusion of steroid receptors [5,6,7].

The present article aims to assess the multitude of AR signaling aspects, encompassing the biological structure of AR, current AR-targeted therapies, mechanisms driving resistance to AR, and AR crosstalk with other pathways, in an attempt to provide a comprehensive and unique document for the PCa research community.

## 2. AR Gene and AR Expression in Prostate Cancer and the Microenvironment 

**Figure 1:**
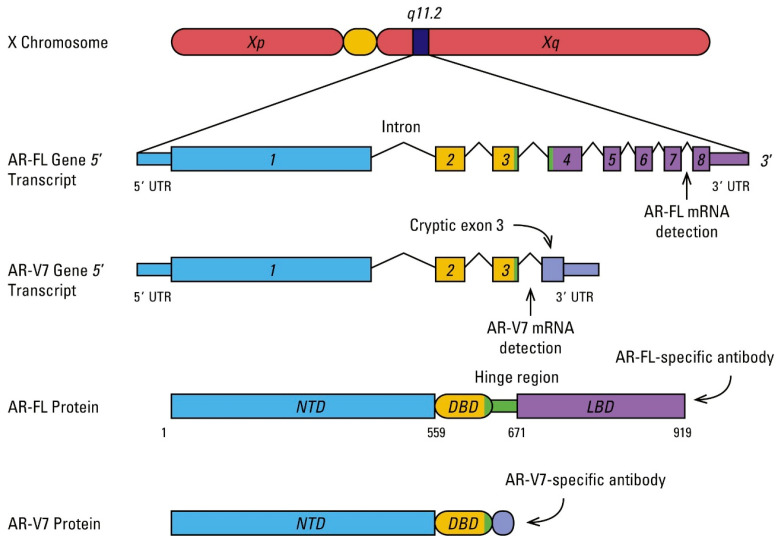
Transcript structures for full-length androgen receptor (AR) and the splice variant AR-V7. Peptide positions are marked according to GRCh36/hg18 human genome sequences (not drawn to scale). Reproduced, with permission, from Luo J. Development of AR-V7 as a Putative Treatment Selection Marker for Metastatic Castration-Resistant Prostate Cancer. *Asian J. Androl.* **2016**, *18*, 580–585; Licensed under a Creative Commons Attribution 4.0 Unported License (Available at https://creativecommons.org/licenses/by/4.0/). Abbreviations: AR-FL, full-length androgen receptor; DBD, DNA-binding domain; LBD, ligand-binding domain; mRNA, messenger RNA; NTD, N-terminal domain; UTR, untranslated region.

The androgen receptor gene codes for a protein of 110 kD composed of 919 amino acids that, similarly to other steroid receptors, is characterized by a conserved DNA-binding domain (DBD) and androgen-binding domain (ABD) and a less conserved N-terminal transactivation domain that contains two polymorphic trinucleotide repeat segments. These repeated segments, composed of a variable number of polyglutamine and polyglycine repeats, influence the transcriptional activity of AR. The full-length gene transcript comprehends eight exons: exon 1 codes for the N-terminal domain, exons 2 and 3 code for the DBD, and exons 4 to 8 code for the ABD [8,9] (Figure 1).

AR expression has been found in almost all primary and metastatic PCa, regardless of stage or grade. AR expression is preserved in the majority of androgen-independent PCa and CRPCa. AR signaling remains active and supports the survival and growth of PCa cells. Intense nuclear AR staining in CRPCa bone metastases is related to worse outcome [10,11]. The prognostic value of AR expression levels determined by immunohistochemistry (IHC) in primary PCa has been inconsistently related to patient outcome, with 20% of studies reporting high AR expression associated with good outcome, 26% showing association with poor outcome, and the others showing no relationship [12].

The crosstalk between the PCa microenvironment and AR signaling is complex, with both pro-tumorigenic and anti-tumorigenic roles observed [13]. Stromal cells express AR and are involved in prostate development and prostate carcinogenesis. It has been demonstrated that before and during prostatic development, the expression of AR in prostate epithelium is undetectable, while the stromal cells express high levels of AR [14,15]. Contrarily to AR expression in PCa cells, low stromal AR expression was significantly associated with biochemical relapse and high-risk clinical parameters, disease progression, and/or poor outcome [16,17].

During the transition from benign to cancerous, stromal cells undergo structural and genomic modification with a progressive decrease in AR expression. Moreover, AR stromal expression decreases linearly from low-grade PCa, becoming almost completely absent in the highest grades [18]. AR expression in malignant prostatic epithelium is less than that in normal/atrophic and hyperplastic glands, supporting the fact that the expression of the receptor decreases in both cellular compartments as the tissues dedifferentiated, with a more pronounced depletion in stromal cells [18]. Metastatic specimens show lower AR stromal expression compared to the corresponding primary PCa. AR stromal expression is further decreased in CRPCa compared to hormone-sensitive PCa [19]. The mechanism behind the loss of AR expression in the stromal cells is yet unknown. The AR signaling in stromal cells targets different genes compared to that in epithelial cells. The PCa cancer cells during carcinogenesis shift from an androgen-dependent paracrine pathway to an autocrine pathway, becoming no longer dependent on stromal–epithelial interactions for their growth and survival [20,21].

## 3. AR Mutations, Amplifications, Splice Variants, and Association with Resistance

Mutations and amplifications of the AR gene are reported in 1% of primary PCa [22] and in about 60% of metastatic tumors [23,24,25]. Metastatic PCa patients treated with AR antagonists have a higher incidence of mutations compared to PCa patients treated with androgen deprivation therapy (ADT) alone [26]. Most AR mutations cause single-amino-acid substitution, predominantly limited to the AR androgen-binding domain [27,28]. The functional consequences of these mutations result in the ability of antiandrogens to function as AR agonists, thus providing a great advantage in terms of cancer progression. The most frequent example is the mutation T877A, reported in around 30% of metastatic CRPCa cases [24,29,30]. Moreover, this mutation enables the activation of AR by other adrenal androgens such as progesterone [31,32], dehydroepiandrosterone (DHEA) [33], and androstenediol [34]. This observation may explain the failure of castration therapy in patients with tumors harboring AR mutations. Specific AR germline polymorphisms have been associated with an increased risk of developing prostate cancer, up to six-fold higher compared to the general population (i.e., R725L) [35]. Other mutations have demonstrated increased binding of AR with co-regulators, thus increasing AR-transcriptional activity (i.e., H874Y [36], W435L [37] mutations). Other mutations seem to confer resistance to enzalutamide and ARN-509 (i.e., F876L [38,39]) (Table 1).

AR amplifications are more frequently found in treated PCa and CRPCa (20–30%) compared to untreated primary PCa. Amplification of the wild-type sequence increases the sensitivity of prostate cells to the reduced level of androgens, allowing PCa to grow even in a low-androgen milieu. It is still not clear whether increased AR gene expression corresponds to increased protein expression and to increased expression of AR target genes [45,46].

Androgen deprivation therapy (ADT) interferes with androgen production and/or is based on antiandrogens that bind the C-terminal ABD of the AR, competitively blocking the activation of the AR by androgens. Other drugs have been developed to target the N-terminal domain (NTD) and the DBD. The first inhibitor of AR’s NTD was EPI-001, which acts by binding covalently to the NTD and so preventing the protein–protein interactions required for transcriptional activity of the AR [47]. EPI-001 is a mixture of four stereoisomers that binds to the activation function-1 (AF-1) region in the NTD of the AR, which is responsible for the AR’ s transcriptional activity.

A phase I study on oral EPI-506, one of the four stereoisomers of EPI-001, for patients with Metastatic Castration-Resistant Prostate Cancer was terminated early due to excessive high pill burden (18 capsules/day) (NCT02606123). DBD-targeting molecules (such as Vancouver Prostate Center (VPC) compounds) are small agents that bind a surface exposed pocket of the DBD, thus interfering with AR–DNA interactions. These compounds are currently under clinical investigation [48]. EPI-7386 is a more potent and stable NTD inhibitor that has been demonstrated to control tumor growth and induce tumor regressions in several CRPC xenografts, including enzalutamide resistant models. It is planned to enter phase I clinical trials in 2020 [49]. 

During the last decade, other mechanisms of AR alterations have been investigated to better understand why some patients are responsive, while others become or are resistant to androgen ablation therapy. Several AR splice variants have been identified in CRPCa cell lines [50]. Some of them lack the ligand-binding domain, but retain their ability to bind DNA in the absence of androgens, thus displaying constitutive activity. AR-V7 was the only variant endogenously detected at the protein level and the most consistently expressed variant in recurrent PCa cell lines and CRPCa specimens [51,52,53] (Figure 1).

During mRNA splicing, AR variants can also originate from exon skipping, like the case of AR^v567es^ that lacks exons 5–7, coding for a portion of the LBD. Similar to AR-V7, AR^v567es^ is constitutively active and can be translated into protein, even though a specific antibody is not available yet. Since multiple studies have confirmed AR^v567es^ expression in PCa specimens, AR^v567es^ has been proposed as another AR variant-dependent mechanism of resistance [54,55,56,57,58]. These two variants, AR-V7 and AR^v567es^, exhibit different transcriptional activity when compared to full-length AR. In particular, AR-V7 was demonstrated to induce expression of genes related to cell cycle progression, including the UBE2C gene [59], a transcription factor that regulates the expression of genes involved in G1/S and G2/M transition and M phase progression [60]. Both AR variant mRNAs have been found in benign prostate tissue, hormone-naïve PCa, and CRPCa samples, with higher levels in the CRPCa subgroup compared to the hormone-naïve [54,61]. The prognostic value of AR-V7 is emphasized by the fact that its overexpression has been associated with an increased risk of biochemical disease recurrence after radical prostatectomy in hormone-naïve prostate cancer patients and with shorter survival in CRPCa [58,62].

## 4. AR Alteration Detection 

AR amplifications can be detected by fluorescence in situ hybridization (FISH), either on tissue or on circulating tumor cells (CTCs) [63]. AR amplifications by circulating DNA have proved to be strongly prognostic in multiple prospective/retrospective cohorts [64,65,66,67].

AR-V7 detection can be achieved via real-time polymerase chain reaction (RT-PCR) on RNA extracted from prostate cancer formalin fixed paraffin embedded (FFPE) samples, CTCs, plasma exosomes, whole blood, and peripheral blood mononuclear cells [58,68,69]. Of interest is the study conducted by Zhu et al. on the evaluation of AR-V7 in 63 prostate tumor biopsies by a junction-specific AR-V7 RNA in situ hybridization (RISH) assay [70]. Higher AR-V7 expression detected and quantified using this highly specific method correlated with poorer response to abiraterone or enzalutamide in mCRPCa.

Serial blood-based analyses of AR-V7 have been performed in patients with metastatic PCa, evidencing temporal changes of AR-V7 expression during AR-target therapies [71]. However, negative AR-V7 patients who converted to positive during therapy accounted for only 14% in the work of Antonarakis and colleagues [72]. Moreover, the prevalence of AR-V7 variants in CRPCa populations varies from 19% [72] to 70% [61], with significant differences among the viable methods of detection. AR-V7 detection by an IHC-based CTC test, the Oncotype Dx AR-V7 Nucleus Detect Test, has also been shown to be predictive in a retrospective study on 142 metastatic CRPCa patients. Patients with AR-V7-positive CTCs benefited from taxane chemotherapy versus AR target inhibitors [73]. This test has received a positive local coverage determination in the United States for patients with CRPCa.

To date, tissue-based assessments of AR expression, amplifications, and detection of splice variants have not been shown to be strongly prognostic or, more importantly, predictive in CRPCa. The paper published in 2020 by the International Society of Urological pathology (ISUP) Working Group recommended that, at present, the evidence is not yet sufficient to justify systematic AR-V7 or AR amplification testing [74].

## 5. Other AR Resistance Mechanisms

As concerns intratumoral androgen biosynthesis, one postulated AR-dependent mechanism deals with the HSD3B1 (Hydroxy-Delta-5-Steroid Dehydrogenase, 3 Beta- And Steroid Delta-Isomerase 1) protein, which commonly generates androstenedione from DHEA precursor in the adrenal gland. The HSD3B1 germline variant is involved in the process of gain-of-function mutations in the LBD and may induce antagonist-to-agonist drug conversion. In particular, point mutations occurring in the AR LBD can redirect molecules with antagonist function into receptor agonists, a process described as most closely associated with AR among hormone nuclear receptors [75]. Such a resistance mechanism may occur for the androgen receptor signaling inhibitor (ARSi) abiraterone acetate and enzalutamide [76]. Again, increased AR activity has been demonstrated to be able to induce drug resistance. There is evidence that in CRPCa, the overexpression of an AR upstream enhancer may stimulate AR elicitation [77].

Regarding AR-independent resistance, a mechanism inducing adaptive resistance is known as “lineage switching”. Under ARSi pressure, prostate cancer cells may acquire different phenotypes contributing to drug resistance; in particular, neuroendocrine PCa differentiation may occur in approximately 25% of metastatic PCa patients. Some data support that ADT can strongly contribute to inducing a neuroendocrine transition [78]. Such a phenotype comprising modifications such as AR downregulation (low or absent AR protein expression) and the acquisition of tumor cells with stem-like habits is notoriously correlated with very poor survival outcomes. Very recently, Sànchez and colleagues showed that the over-induction of AMP-activated kinase (AMPK) is correlated with neuroendocrine and stemness properties reduction [79]. Of interest, a recent large prospective study of patients progressing under abiraterone acetate/enzalutamide underlined that different tumor features, such as neuroendocrine markers, loss of the retinoblastoma susceptibility gene (*RB1*), small cell histology, and AR expression modulation, have only partial connections [80]. Another AR-independent mechanism includes “bypass signaling”, in which a protein not involved in the AR pathway signaling may induce the transcription of AR-related genes. This process does not trigger AR signaling, being completely independent, and a driver of this is believed to be enhanced glucocorticoid receptor activity [75].

## 6. New Approvals in Non-mCRPCa

Three pivotal ARSi trials—enzalutamide, apalutamide, and darolutamide—have each demonstrated metastasis-free survival (MFS) benefits in patients with non-metastatic castration-resistant PCa (non-mCRPCa).

In the double-blind, randomized (2:1 ratio), phase 3 PROSPER trial (i.e., Enzalutamide in Men with Non-metastatic, Castration-Resistant Prostate Cancer), a total of 1401 non-mCRPCa subjects with increasing prostate-specific antigen (PSA) values (PSA doubling time of ≤10 months) were treated with enzalutamide at a dose of 160 mg or placebo once daily. All the patients continued to receive ADT concurrently. The enzalutamide arm was correlated with greater benefit in terms of median MFS over the placebo arm (36 months versus 14 months, respectively), with a 71% risk reduction in metastasis or death (*p* < 0.001). A subsequent antineoplastic therapy was significantly delayed with enzalutamide over placebo (39 versus 17 months, respectively (*p* < 0.001)), as was the time to PSA progression (37 months versus 3 months, respectively (*p* < 0.001)), and a lower rate of disease progression was observed with enzalutamide (22% versus 69%) [81]. More recently, survival data from the preplanned third interim analysis underlined that enzalutamide was associated with longer median overall survival (OS) than placebo (67 months versus 56 months, respectively (*p* = 0.001)), along with a 27% reduction in the risk of death (*p* = 0.001). Interestingly, both the treatment arms had similar rates of adverse events [82].

The phase 3 randomized (2:1 ratio), double-blind, SPARTAN (Selective Prostate Androgen Receptor Targeting with ARN-509) trial assessed the ARSi apalutamide at a dose of 240 mg daily plus ADT against placebo plus ADT in 1207 patients with non-mCRPCa having a PSA doubling time of ≤10 months. The findings demonstrated that patients in the apalutamide group had longer median MFS than did those in the placebo group (40 months versus 16 months, respectively (*p* < 0.001)), with the risk of metastasis or death reduced by 72% with the study drug. The median time to symptomatic progression was also in favor of apalutamide over placebo (*p* < 0.001). Higher rates of adverse events (rash, hypothyroidism, and fracture) occurred with apalutamide than with placebo; however, this was mainly for grade 1 or 2, and the discontinuation rate was <11% for both groups [83].

The double-blind, randomized (2:1 ratio), phase 3, placebo-controlled ARAMIS (Androgen Receptor Antagonizing Agent for Metastasis-free Survival) trial was designed to prove whether adding the ARSi darolutamide to ADT showed benefits over ADT alone in non-mCRPCa. A total of 1509 men were treated with darolutamide (955 men) or placebo (554 men). With a median follow-up of 29 months, better OS resulted with darolutamide than with placebo (83% versus 77%, respectively), with a significantly reduced risk of death (hazard ratio 0.69, *p* = 0.003). The study drug also produced benefits for all secondary and exploratory endpoints in the intent-to-treat population, among which were the time to pain progression (*p* < 0.001), delaying the initiation of chemotherapy (*p* < 0.001), and the first onset of a symptomatic skeletal event (*p* = 0.005) [84].

## 7. AR inhibition in Metastatic Castration-Sensitive PCa

In the phase 3, double-blind LATITUDE trial (i.e., A Study of Abiraterone Acetate Plus Low-Dose Prednisone Plus Androgen Deprivation Therapy (ADT) Versus ADT Alone in Newly Diagnosed Participants With High-Risk, Metastatic Hormone-Naive Prostate Cancer), a total of 1209 patients with metastatic castration-sensitive PCa (mCSPCa) and at least two out of three risk factors identifying a high-volume disease (Gleason score of ≥8, ≥3 bone metastases by bone scan, or the occurrence of measurable visceral metastases not including lymph node metastases) were screened. A total of 1199 of these patients were finally randomized to abiraterone acetate plus prednisone plus ADT (abiraterone group) versus ADT plus dual placebos (placebo group). The first interim analysis after a median follow-up of 30 months demonstrated a survival benefit in terms of both the median OS and median radiographic progression-free survival, not reached versus 34 months (hazard ratio 0.62), and 33 versus 14 months (hazard ratio 0.47), respectively (*p* < 0.001 for both) [85]. Afterwards, with a median follow-up of 51 months, the final OS analysis confirmed the advantage in favor of abiraterone acetate over the placebo group: 53 months versus 36 months, respectively, with a risk reduction of 34% (*p* < 0.0001). The OS benefit was maintained in all subgroups of patients, except for patients with Eastern Cooperative Oncology Group (ECOG) performance status of 2. All secondary endpoints (PSA progression, pain progression, onset of a skeletal event, start of chemotherapy/initiation of subsequent anticancer therapy) were significantly improved with the study drug. Grade 3 or 4 adverse events occurred in 68% of patients receiving abiraterone acetate versus 50% in the placebo group, leading to treatment discontinuation in 4% versus 2% of patients, respectively. From this, abiraterone acetate plus prednisone is a new standard of care in this disease setting [85].

The phase 3, double-blind, placebo-controlled ARCHES trial (i.e., A Study of Enzalutamide Plus Androgen Deprivation Therapy (ADT) Versus Placebo Plus ADT in Patients With Metastatic Hormone Sensitive Prostate Cancer) evaluated the efficacy and the safety profile of enzalutamide in 1150 mCSPCa patients who were previously stratified by prior chemotherapy with docetaxel and by disease volume. Patients were randomized (1:1 ratio) to enzalutamide plus ADT versus placebo plus ADT. The results showed that the study drug was associated with a lower risk of radiographic progression-free survival or death versus the placebo arm (median not reached versus 19 months, respectively (*p* < 0.001)), with a risk reduction equal to 61%. The benefit of enzalutamide was demonstrated in all preplanned subgroups. This also included patients with low-volume disease and/or previously treated with docetaxel. The incidence of grade ≥3 adverse events was similar between the two treatment arms—24% for enzalutamide and 25% for placebo—and no unexpected side effects occurred. The authors therefore stated that enzalutamide treatment should be indicated as a new option of care in mCSPCa patients [86].

Subsequently, the phase 3 randomized, open-label trial ENZAMET (Enzalutamide in First-Line Androgen Deprivation Therapy for Metastatic Prostate Cancer) demonstrated a significant OS benefit for patients receiving enzalutamide at a dose of 160 mg per day in addition to ADT, as compared to ADT alone, with or without early docetaxel. It should be noted that, especially in patients previously treated with docetaxel, side effects, including seizures, were mostly associated with the enzalutamide arm [87].

Apalutamide was investigated in the phase 3, randomized, double-blind, placebo-controlled TITAN (The Targeted Investigational Treatment Analysis of Novel Anti-androgen) trial. In this trial, 1052 mCSPCa patients received apalutamide at 240 mg a day or placebo, always maintaining ADT. The results showed that the study drug significantly improved both OS and radiographic progression-free survival. Grade 3 or 4 adverse events occurred with similar frequency across the two treatment arms—around 40% [88].

## 8. Ongoing AR-Based Novel Drug Combinations in mCRPCa

Here we report phase 1/2 studies exploring drug strategies encompassing AR-targeted molecules, mostly the ARSi enzalutamide being quite safe regarding its toxicity profile, combined with different drug classes such as poly(adenosine diphosphate [ADP]-ribose) polymerase inhibitors (PARPi), nanoparticle-based drugs, immunotherapeutics, and radioactive iodine, respectively. A phase 1 study of PARPi niraparib with ARSi apalutamide or abiraterone acetate plus prednisone in people with mCRPCa with or without DNA damage repair (DDR) defects has recently been completed [NCT02924766]. Another phase 1 study of the combination of niraparib and enzalutamide in men with mCRPCa has been terminated [NCT02500901]. A phase 2 study currently recruiting patients is testing CRLX101—a nanoparticle drug conjugate with camptothecin—plus enzalutamide in mCRPCa patients progressing under enzalutamide [NCT03531827]. In a phase 1b/2 study, enzalutamide is being included in a treatment arm (arm C) with anti-PD-L1 avelumab and bempegaldesleukin (NKTR-214, a CD122-biased IL-2 receptor agonist) for mCRPCa patients [NCT04052204]. In addition, a randomized phase 2 study is evaluating I-131-1095 radiotherapy in combination with enzalutamide compared with enzalutamide alone in chemotherapy-naïve prostate-specific membrane antigen (PSMA)-avid mCRPCa patients progressing under abiraterone [NCT03939689] (Table 2).

## 9. Recent Data on Anti-AR Combination Therapy across PCa Stages

Investigating AR-targeted therapies continues to be a cornerstone in PCa research, throughout all stages of disease. In very high risk clinically localized PCa patients, the phase 3, double-blind, randomized ANZUP1801 trial (i.e., Darolutamide Augments Standard Therapy for Localised Very High-Risk Cancer of the Prostate) has been planned to investigate the addition of the ARSi darolutamide or placebo to ADT and definitive/salvage radiotherapy; survival data are awaited. At the American Society of Clinical Oncology (ASCO) Virtual Annual Meeting 2020, updated results of the PROSPER, SPARTAN, and ARAMIS trials demonstrated improvements in OS for patients with non-metastatic CRPCa randomized to enzalutamide, apalutamide, and darolutamide, respectively, as compared to ADT alone. A post hoc analysis of the ARCHES trial with stratification by baseline PSA levels was presented at the ESMO (European Society for Medical Oncology) Virtual Annual Meeting 2020. The authors found that the benefit of enzalutamide over placebo in terms of Radiographic progression-free survival (rPFS) was consistent across three strata of baseline PSA values, ≤0.2, 0.2–4, and >4 ng/mL. Preliminary data of the phase 3 IMbassador250 trial presented at the AACR (American Association for Cancer Research) 2020 Virtual Meeting show no survival benefit with the checkpoint inhibitor atezolizumab combined with enzalutamide in mCRPCa patients after disease progression to abiraterone acetate. Of interest, a very recent phase 1 trial of the c-MET (also called tyrosine-protein kinase Met or hepatocyte growth factor receptor) inhibitor crizotinib plus enzalutamide in men with mCRPCa showed a marked reduction in systemic exposure of crizotinib, likely caused by enzalutamide induction of Cytochrome P450 3A4 (CYP3A4) [89]. This evidence, as stated by the authors, underlines the need to investigate pharmacokinetic drug interactions when planning novel treatment strategies. It is known that loss-of-function *PTEN* (Phosphatase And Tensin Homolog) mutations occur in 8% of all PCa patients and correlate with worse outcomes, along with reduced benefit from ARSi molecules. The initial results of the phase 3, double-blind, placebo-controlled, randomized IPATential150 trial) i.e., Ipatasertib plus Abiraterone vs. Placebo plus Abiraterone in Metastatic Castration-Resistant Prostate Cancer) were presented at the most recent ESMO Meeting. A total of 1101 asymptomatic or mildly symptomatic untreated mCRPCa patients were randomized (1:1) to receive ipatasertib plus abiraterone acetate versus abiraterone acetate alone. Patients with PTEN-loss and those who were treated with the combination arm showed a significant improvement in median radiographic PFS, with a risk reduction of 23%.

## 10. Conclusions

During recent decades, several therapies have been developed for PCa treatment, and the therapeutic armamentarium is continuing to increase. AR signaling is the main pathway involved in PCa growth, and accordingly, anti-AR molecules are in development at all stages of PCa, from high-risk localized tumors to mCRPCa stages. Despite the wide use of AR-targeted therapies, biomarker-driven tools capable of orienting therapeutic choices are so far lacking in these patients when considering the drug therapies reported here. This could become a serious embarrassment for clinicians when all the anti-AR therapies become available and are introduced into daily clinical practice. Conversely, according to the last National Comprehensive Cancer Network (NCCN) PCa guidelines, molecular-driven therapy is currently a well-defined approach in mCRPCa patients, both for the PARPi olaparib with homologous repair gene mutated tumors and for the immune checkpoint blocker pembrolizumab when MicroSatellite Instability High (MSI-H) or MisMatch Repair deficiency (dMMR) does occur.

In the near future, one dangerous risk from the extensive use of therapies targeting the AR axis could be the increase in AR-independent tumors, notoriously associated with worse prognosis. In the era of precision medicine, the main question for the clinician is “what is the best treatment option for my patient?” The continuously improving diagnostic techniques, along with the increasing amount of data emerging from clinical trials, will allow a better understanding of the molecular characteristics of the tumor and their predictive significance for treatment resistance or sensitivity.

## Figures and Tables

**Table 1:** Most common androgen receptor (AR) mutations in prostate cancer and associations with drug resistance.

AR Point Mutations	Associated Drug Resistance	References
F877L	Enzalutamide Apalutamide	Balbas 2013 [40] Joseph 2013 [39] Korpal 2013 [38]
T878A	Abiraterone	Cai 2011 [41] Chen 2015 [42]
W742C	Bicalutamide	Hara 2003 [43]
V716T	Flutamide	Hara 2003 [43]
H875Y	Flutamide Abiraterone	Azad 2015 [44] Taplin 1999 [26]

**Table 2:** Ongoing trials on AR-based novel drug combinations.

NCT	Phase	Drug	Patients
NCT02924766	1	Niraparib with ARSi apalutamide or abiraterone acetate plus prednisone	mCRPCa with or without DDR defects
NCT02500901	1	Niraparib and enzalutamide	mCRPCa
NCT03531827	2	CRLX101—a nanoparticle drug conjugate with camptothecin—plus enzalutamide	mCRPCa
NCT04052204	1b/2	Treatment arm C: Enzalutamide with Avelumab and bempegaldesleukin (NKTR-214, a CD122-biased IL-2 receptor agonist)	mCRPCa
NCT03939689	2	I-131-1095 radiotherapy in combination with enzalutamide compared with enzalutamide alone	chemotherapy-naïve PSMA-avid mCRPCa

## Abbreviations

ABD	androgen-binding domain
ADT	androgen deprivation therapy
AMPK	AMP-activated kinase
AR	androgen receptor
ARE	androgen response element
DBD	DNA-binding domain
DDR	DNA damage repair
DHEA	dehydroepiandrosterone
DHT	5α-dihydrotestosterone
ECOG	Eastern Cooperative Oncology Group
CSPCa	castration-sensitive prostate cancer
PCa	prostate cancer
PSMA	prostate-specific membrane antigen
NE	neuroendocrine
CRPCa	castration-resistant prostate cancer
PSA	prostate-specific antigen
